# TRIM28 Promotes Keratinocyte Proliferation and Invasion by Activating NLRP3/SGT1 Axis‐Mediated Macrophage Pro‐Inflammatory Polarization in an In Vitro Macrophage–Keratinocyte Co‐Culture Model

**DOI:** 10.1002/iid3.70522

**Published:** 2026-09-10

**Authors:** Zhe Gao, Xin Zhang, Juan Wang, Ni Yang

**Affiliations:** ^1^ Department of Dermatology Hangzhou Third People's Hospital Hangzhou Zhejiang China

**Keywords:** in vitro co‐culture, keratinocyte proliferation, macrophage polarization, NLRP3 inflammasome, TRIM28

## Abstract

**Background:**

Macrophage–keratinocyte crosstalk is an important component of cutaneous inflammatory responses. Psoriasis is a chronic inflammatory skin disease characterized by dysregulated keratinocyte proliferation and immune dysfunction. While macrophage pro‐inflammatory activation is known to exacerbate psoriatic inflammation, the underlying molecular mechanisms remain incompletely understood. TRIM28, a multifunctional regulatory protein, may play a role in this process, but whether TRIM28 regulates macrophage–keratinocyte crosstalk in a psoriasis‐relevant inflammatory context remains unclear. The present study was designed as an in vitro mechanistic investigation and does not establish TRIM28 dysregulation in human psoriasis. Therefore, psoriasis is discussed only as a potential disease context, rather than as a conclusion directly supported by the present data.

**Methods:**

Human THP‐1 monocytes were differentiated into macrophages and subjected to TRIM28 knockdown or overexpression. Macrophage polarization status was evaluated using a limited panel of conventional M1‐like/M2‐like‐associated markers via flow cytometry (CD68/CD86/CD206), cytokine secretion (ELISA for IL‐6, TNF‐α, IL‐10), and marker expression (iNOS/Arg1 by Western blot). The NLRP3/SGT1 axis and NLRP3 SUMOylation were evaluated using Western blot and co‐immunoprecipitation, respectively. Inflammasome‐associated activation was further assessed by detecting cleaved caspase‐1 and mature IL‐1β expression using Western blot and secreted IL‐1β using ELISA. The interaction between TRIM28 and NLRP3 was examined by co‐immunoprecipitation. Functional impacts on keratinocytes were examined using a Transwell co‐culture system with HaCaT cells, measuring proliferation (CCK‐8) and invasion (Transwell assays). Rescue experiments involved NLRP3/SGT1 knockdown or SUMOylation inhibition (2‐D08).

**Results:**

TRIM28 overexpression promoted macrophage M1‐like/pro‐inflammatory marker expression, increased pro‐inflammatory cytokine secretion, and elevated NLRP3 and SGT1 expression, while enhancing NLRP3 SUMOylation. TRIM28 overexpression also increased cleaved caspase‐1, mature IL‐1β, and IL‐1β secretion, whereas TRIM28 knockdown exerted the opposite effects. Co‐immunoprecipitation further showed an interaction between TRIM28 and NLRP3. Conversely, TRIM28 knockdown induced a shift toward an M2‐like/anti‐inflammatory marker profile and reduced inflammation. In co‐culture, TRIM28‐overexpressing macrophages significantly enhanced HaCaT cell proliferation and invasion. These effects were reversed by NLRP3/SGT1 silencing or SUMOylation inhibition, supporting the involvement of the NLRP3/SGT1 axis and NLRP3 SUMOylation in TRIM28‐mediated actions.

**Conclusion:**

TRIM28 promotes macrophage pro‐inflammatory polarization‐like changes by activating the NLRP3/SGT1 axis via NLRP3 SUMOylation, subsequently promoting keratinocyte proliferation and invasion in an in vitro macrophage–keratinocyte co‐culture system. These findings suggest a potential mechanism relevant to inflammatory macrophage–keratinocyte interactions, but validation in psoriatic tissues, animal models, and clinical datasets is required before TRIM28 can be implicated in psoriasis pathogenesis. Accordingly, the present study should be interpreted as a basic mechanistic investigation rather than clinical or disease‐level evidence linking TRIM28 to psoriasis.

## 1 Introduction

Psoriasis is a chronic, immune‐mediated inflammatory skin disorder that affects approximately 2%–3% of the global population, imposing significant physical, psychological, and socioeconomic burdens on patients worldwide [1]. This multisystem disease not only manifests as erythematous, scaly plaques on the skin but is also associated with various comorbidities, including depression, metabolic syndrome, and cardiovascular diseases, substantially reducing patients' quality of life [2]. Over the past decade, major advances in biologic therapy have substantially improved the management of moderate‐to‐severe psoriasis [3]. Agents targeting IL‐23, IL‐17, TNF‐α, and related pathways can achieve high levels of skin clearance in a large proportion of patients and have reshaped the current therapeutic landscape [4]. Nevertheless, psoriasis remains a chronic relapsing disease, and some patients may experience incomplete response, secondary loss of response, contraindications, limited access, treatment discontinuation, or recurrence after withdrawal [5]. Therefore, continued investigation of inflammatory circuits that regulate keratinocyte–immune cell interactions remains relevant, particularly for understanding disease biology and identifying complementary mechanistic targets.

The pathogenesis of psoriasis is driven by complex interactions among innate immune cells, adaptive immune cells, and keratinocytes [6]. Dendritic cells are key initiators and amplifiers of psoriatic inflammation through the production of cytokines such as IL‐23, IL‐12, and TNF‐α, which promote the differentiation and maintenance of pathogenic Th17 and Th1 responses [7]. The IL‐23/IL‐17 axis is central to psoriasis pathogenesis: IL‐23 sustains Th17 cells and other IL‐17‐producing immune populations, while IL‐17A, IL‐17F, and IL‐22 act on keratinocytes to induce hyperproliferation, altered differentiation, chemokine release, and antimicrobial peptide production [8]. In addition, IL‐36 family cytokines produced by keratinocytes and immune cells further amplify cutaneous inflammation by enhancing dendritic cell activation, neutrophil recruitment, and cytokine production [9]. These interconnected pathways form a self‐sustaining inflammatory loop between immune cells and keratinocytes.

Macrophage activation and phenotypic plasticity play important roles in the pathogenesis of psoriasis by exacerbating inflammation and immune dysregulation. Psoriasis is characterized by abnormal keratinocyte proliferation and inflammatory cell infiltration, with pro‐inflammatory macrophage states being potential contributors to disease progression [10]. Macrophage polarization is now recognized as a dynamic and context‐dependent spectrum rather than a strict M1/M2 dichotomy. Nevertheless, the M1‐like/M2‐like terminology remains a commonly used operational framework in in vitro studies to describe macrophages with relatively pro‐inflammatory or anti‐inflammatory marker profiles. M1‐like macrophages, classically activated by stimuli such as LPS and IFN‐γ, produce substantial reactive oxygen species and inflammatory mediators, including IL‐23, TNF‐α, IL‐6, and IL‐1β, which perpetuate oxidative stress and T‐cell dysregulation, further aggravating psoriatic lesions [11]. Conversely, M2‐like macrophages are generally associated with tissue repair and anti‐inflammatory responses, although these categories do not represent fixed macrophage lineages. The imbalance between pro‐inflammatory and anti‐inflammatory macrophage states is observed in inflammatory skin diseases, where pro‐inflammatory macrophage activation can drive inflammatory responses and inhibit tissue repair processes [12]. Notably, pro‐inflammatory macrophages enhance the recruitment of inflammatory T cells, particularly Th1 and Th17 subsets, through the secretion of cytokines like IL‐12 and IL‐23, thereby sustaining the inflammatory cascade in psoriasis [13]. The identification of specific biomarkers for pro‐inflammatory macrophage activation, such as interferon‐responsive genes (ISG15, RSAD2, IFIT3), further underscores the importance of M1 macrophages in psoriasis and provides potential targets for therapeutic intervention [14]. Although macrophages are not the sole drivers of psoriasis, they contribute to inflammatory cytokine production and may interact with keratinocytes within the broader IL‐23/IL‐17‐, IL‐36‐, and TNF‐related inflammatory network [15]. Thus, macrophage–keratinocyte crosstalk represents one relevant cellular process for mechanistic investigation, while requiring careful interpretation when studied in simplified in vitro systems.

TRIM28 (tripartite motif‐containing 28), a multifunctional protein involved in transcriptional regulation and epigenetic modifications, has emerged as a potential regulator of immune responses and inflammatory diseases [16]. TRIM28 modulates protein stability and signaling pathways through ubiquitination and SUMOylation, influencing diverse biological processes such as immune responses, cell cycle progression, and stress responses [17]. Currently, the role of TRIM28 in psoriasis remains unclear. Importantly, direct evidence showing TRIM28 dysregulation in human psoriatic lesions or its correlation with clinical disease activity is still lacking. Therefore, the present study does not aim to establish TRIM28 as a confirmed psoriasis‐associated molecule in vivo. Instead, we investigated whether TRIM28 regulates macrophage polarization and macrophage‐mediated keratinocyte proliferation and invasion in a simplified in vitro co‐culture system. We also acknowledge that the present study does not position TRIM28 within validated psoriasis‐defining pathways such as the IL‐23/IL‐17 axis, IL‐36 signaling, or dendritic cell‐driven immune activation. Rather, our work focuses on TRIM28‐dependent regulation of the NLRP3/SGT1 axis and macrophage polarization as a basic inflammatory mechanism that may be relevant to macrophage–keratinocyte communication. Given the limited marker panel used in this study, macrophage phenotypes are described as M1‐like or M2‐like marker shifts rather than definitive polarization states. Thus, the rationale of this study is not to prove a psoriasis‐specific role of TRIM28, but to define a cell‐line‐based mechanism by which TRIM28 may influence inflammatory macrophage–keratinocyte interactions. Any disease‐level relevance to psoriasis remains hypothetical and requires additional validation in human tissues, patient‐derived samples, transcriptomic datasets, and in vivo models.

## 2 Materials and Methods

### 2.1 Cell Culture and Differentiation

The human monocytic cell line THP‐1 was obtained from iCell (iCell‐h213, China) and maintained in RPMI‐1640 medium (GNM31800‐5, Genomcell, China) supplemented with 10% fetal bovine serum (FBS) and 1% penicillin‐streptomycin at 37°C in a humidified incubator with 5% CO_2_. To differentiate THP‐1 cells into macrophages, cells were treated with 100 nM phorbol 12‐myristate 13‐acetate (PMA, S1819‐1mg, Beyotime) for 24 h. HaCaT keratinocytes were obtained from iCell (iCell‐h066, China) and cultured in high glucose DMEM (GNM12800‐2, Genomcell, China) with 10% FBS and 1% penicillin‐streptomycin.

### 2.2 RNA Interference and Plasmid Transfection

For gene knockdown, small interfering RNAs (siRNAs) targeting TRIM28 (si‐TRIM28#1, si‐TRIM28#2, and si‐TRIM28#3), NLRP3 (si‐NLRP3), and SGT1 (si‐SGT1), along with a negative control siRNA (si‐NC), were designed and synthesized by GenePharma (China). Macrophages were transfected with 50 nM siRNA using Lipofectamine 3000 reagent (L3000‐008, Invitrogen, USA) according to the manufacturer's instructions. Sequences of siRNAs are shown in Table 1. For overexpression, the full‐length human TRIM28 coding sequence was cloned into a pcDNA3.1 vector (TRIM28 OE, GenePharma, China). The empty vector served as the negative control (OE‐NC). Lentiviral particles containing vectors were prepared and transfected using Lipofectamine 3000 reagent (L3000‐008, Invitrogen, USA) according to the manufacturer's instructions. Cells were harvested 48 h post‐transfection for subsequent analyses.

**Table 1:** Sequences of siRNAs.

Gene	Sense (5′–3′)	Antisense (5′–3′)
si‐TRIM28#1	CACUAGCUGUGAGGAUAAUTT	AUUAUCCUCACAGCUAGUGTT
si‐TRIM28#2	GGCGAGAUGAAGUUUCAGUTT	ACUGAAACUUCAUCUCGCCTT
si‐TRIM28#3	GCAACGUACACAAGCAUGATT	UCAUGCUUGUGUACGUUGCTT
si‐NLRP3	GCCAGCUGGAAUUGUUCUATT	UAGAACAAUUCCAGCUGGCTT
si‐SGT1	ACAAUUCGUAGCAGAUGUATT	UACAUCUGCUACGAAUUGUTT

### 2.3 Western Blotting

Total protein was extracted using RIPA lysis buffer (P0013B, Beyotime, China) containing protease inhibitors. Protein concentration was measured using a BCA assay kit (P0012, Beyotime, China). Equal amounts of protein (20 µg per lane) were separated by 12% SDS‐PAGE and transferred onto PVDF membranes. Membranes were blocked with 5% non‐fat milk for 1 h at room temperature, followed by overnight incubation with primary antibodies against TRIM28 (1:3000, DF7531, Affinity, USA), iNOS (1:1000, ab3523, Abcam, USA), Arg1 (1:2000, DF6657, Affinity, USA), cleaved caspase‐1 (1:2000, AF4005, Affinity, USA), IL‐1β (1:2000, AF5103, Affinity, USA), NLRP3 (1:2000, DF7438, Affinity, USA), SGT1 (1:2000, DF12481, Affinity, USA), and GAPDH (1:10,000, 10494‐1‐AP, Proteintech, USA) at 4°C. After washing, membranes were incubated with HRP‐conjugated secondary antibodies (1:6000, 7074, CST, USA). Protein bands were visualized using enhanced chemiluminescence and analyzed with ImageJ software.

### 2.4 Co‐Immunoprecipitation (Co‐IP)

To assess the SUMOylation level of NLRP3 in macrophages, cells were lysed in RIPA buffer (P0013B, Beyotime, China) supplemented with PMSF (P0100, Solarbio, China), a phosphatase inhibitor cocktail (P1260, Solarbio, China), and a protease inhibitor mixture (P6730, Solarbio, China). After protein quantification with a BCA kit (P0012S, Beyotime, China), lysates were incubated with primary antibodies against NLRP3 (68102‐1‐Ig, Proteintech, USA) overnight at 4°C, followed by incubation with Protein A/G agarose beads from an IP/Co‐IP kit (C600689‐0020, BBI Life Sciences, USA). Immunoprecipitates were separated by SDS‐PAGE using a 10% gel prepared with 30% acrylamide (B546017‐0500, Sangon Biotech, China) and transferred to a PVDF membrane (10600023, GE Healthcare Life Sciences, USA). Membranes were blocked with 5% BSA (4240GR100, Biofroxx, Germany) and probed with primary antibodies against SUMO1 (10329‐1‐AP, Proteintech, USA), NLRP3 (68102‐1‐Ig, Proteintech, USA), TRIM28 (DF7531, Affinity, USA), and β‐actin (81115‐1‐RR, Proteintech, USA), followed by HRP‐conjugated secondary antibodies (7074, CST, USA). Signals were developed with ECL Plus substrate (PE0010, Solarbio, China) and analyzed with ImageJ. To further examine the association between TRIM28 and NLRP3, cell lysates were immunoprecipitated with anti‐TRIM28 antibody and immunoblotted with anti‐NLRP3 antibody, with IgG used as a negative control.

### 2.5 Flow Cytometry

Macrophage polarization was evaluated by surface marker staining. Cells were harvested and incubated with FITC‐conjugated anti‐CD68 (333805, Biolegend, USA) and APC‐conjugated anti‐CD86 (374207, Biolegend, USA) as a conventional M1‐like/pro‐inflammatory‐associated marker combination or PE‐conjugated anti‐CD206 (321105, Biolegend, USA) (for M2) antibodies at 4°C for 30 min. After washing, cells were analyzed using a flow cytometer (NovoCyte, Agilent, USA). Data were processed with FlowJo software. These markers were used to indicate relative phenotypic shifts and were not intended to define fixed macrophage lineages.

### 2.6 ELISA

Levels of IL‐6 (RX103064H), IL‐1β (RX106152H), TNF‐α (RX104793H), and IL‐10 (RX106126H) in cell culture supernatants were measured using commercially available ELISA kits (Ruixin Biotech, China) according to the manufacturer's instructions. Absorbance was read at 450 nm using a microplate reader (CMaxPlus, MD, USA).

### 2.7 Co‐Culture System

Macrophages (1 × 10^5^ cells) were seeded into the upper chamber of a Transwell insert (0.4 µm pore size), while HaCaT cells (1 × 10^5^ cells) were placed in the lower chamber. Cells were co‐cultured in DMEM with 10% FBS for 24 h. HaCaT cells were then collected for functional assays.

### 2.8 CCK‐8 Assay

The viability of HaCaT cells was assessed using the CCK‐8 (C0039, Beyotime, China). Cells were seeded into 96‐well plates at a density of 15,000 cells per well and incubated overnight. After treatment according to the experimental groups, cells were incubated with CCK‐8 reagent for the designated time. Absorbance was measured at 450 nm using a microplate reader (CMaxPlus, MD, USA). Cell viability was calculated as (experimental absorbance − blank absorbance)/(control absorbance − blank absorbance) × 100%.

### 2.9 Cell Invasion Assay

The invasive ability of HaCaT cells was evaluated using Transwell chambers. The upper chambers were pre‐coated with 30 μL of a 3:1 dilution of Basement Membrane Matrix Matrigel (082724, Jimo Biotechnology, China) and incubated overnight at 4°C. After treatment, cells were seeded into the upper chambers with serum‐free medium, while complete medium was added to the lower chamber. Following 24 h of incubation, non‐invading cells on the upper membrane surface were removed with a cotton swab. Invaded cells were fixed with 4% paraformaldehyde (30525‐89‐4, Maklin, China) for 10 min, stained with 0.1% crystal violet for 30 min, and photographed under an inverted microscope (Ts2‐FC, Nikon, Japan). The number of invaded cells from three random fields was counted using ImageJ software.

### 2.10 Statistical Analysis

All experiments were performed in triplicate. Data are presented as mean ± standard deviation. Differences between groups were analyzed using one‐way ANOVA followed by Tukey's post hoc test. A *p* value < 0.05 was considered statistically significant. All analyses were performed using GraphPad Prism 10.0.

## 3 Results

### 3.1 TRIM28 Modulated Macrophage Polarization

**Figure 1:**
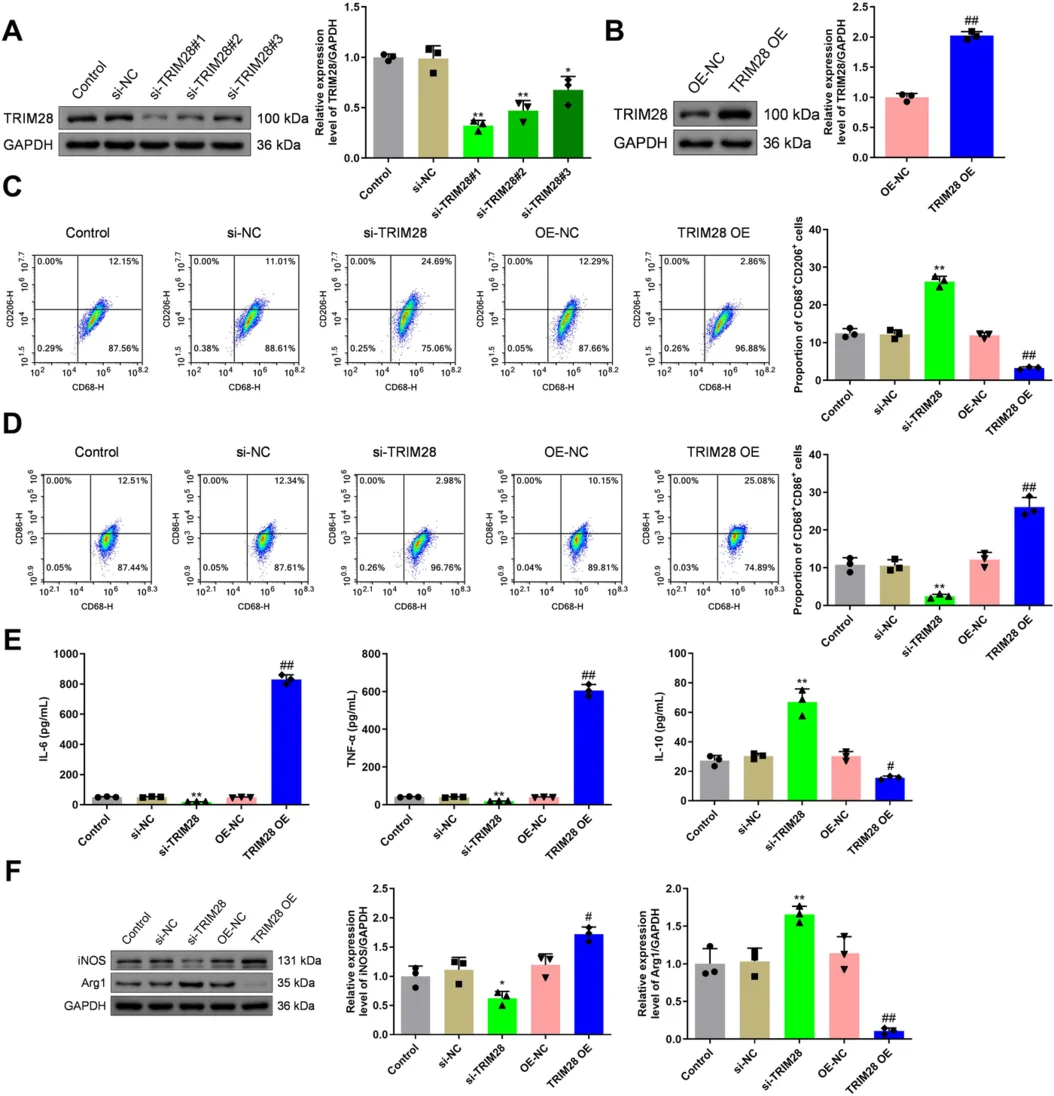
TRIM28 regulated macrophage phenotypic polarization and inflammatory cytokine secretion. (A) Western blot analysis of TRIM28 protein expression in macrophages transfected with three specific si‐TRIM28 sequences (si‐TRIM28#1, si‐TRIM28#2, si‐TRIM28#3) or negative control (si‐NC), with untransfected cells as Control. (B) Western blot analysis of TRIM28 protein expression in macrophages transfected with TRIM28 overexpression plasmid (TRIM28 OE) or empty vector control (OE‐NC). (C) Flow cytometry analysis of CD68^+^CD206^+^ M2‐like macrophage proportions. (D) Flow cytometry analysis of CD68^+^CD86^+^ M1‐like macrophage proportions. (E) ELISA measurements of pro‐inflammatory cytokines (IL‐6, TNF‐α) and anti‐inflammatory cytokine (IL‐10) secretion levels in macrophage culture supernatants. (F) Western blot analysis of the M1‐like‐associated marker (iNOS) and the M2‐like‐associated marker (Arg1) protein expressions, with GAPDH as the loading control. These markers were used to indicate relative phenotypic shifts rather than definitive macrophage subtypes. *p < 0.05; **p < 0.01 versus si‐NC; ^#^ *p* < 0.05; ^##^ *p* < 0.01 versus OE‐NC.

To investigate the role of TRIM28 in macrophage polarization and subsequent effects on HaCaT cells, we first constructed TRIM28 knockdown and overexpression macrophage models. THP‐1 cells were induced to differentiate into macrophages with 100 nM PMA for 24 h, followed by transfection with si‐TRIM28 (three specific sequences) or TRIM28 overexpression plasmid (TRIM28 OE) for 48 h, with si‐NC and OE‐NC as negative controls, respectively. The expression level of TRIM28 in macrophages transfected with si‐TRIM28#1, si‐TRIM28#2, and si‐TRIM28#3 was markedly decreased, with the lowest expression observed in TRIM28#1‐transfected macrophages (Figure 1A). Thus, TRIM28#1 was utilized to silence TRIM28 in subsequent experiments. In contrast, TRIM28 expression in the TRIM28 OE group was remarkably upregulated compared with the OE‐NC group (Figure 1B), confirming the effective establishment of TRIM28 overexpression macrophages.

To determine the effect of TRIM28 on macrophage polarization, we detected the phenotypic markers, inflammatory cytokine secretion, and key molecular expressions in TRIM28‐modified macrophages. Flow cytometry analysis revealed that TRIM28 knockdown (si‐TRIM28 group) significantly increased the proportion of CD68^+^CD206^+^ M2‐like macrophages to 25% compared with the si‐NC group, while TRIM28 OE reduced the proportion to 5% (Figure 1C). Conversely, the proportion of CD68^+^CD86^+^ M1‐like macrophages was decreased to 5% in the si‐TRIM28 group but increased to 25% in the TRIM28 OE group (Figure 1D). ELISA results showed that TRIM28 knockdown downregulated the secretion of pro‐inflammatory cytokines IL‐6 and TNF‐α while upregulating the anti‐inflammatory cytokine IL‐10, whereas TRIM28 overexpression exerted the opposite effects (Figure 1E). Western blot analysis further confirmed that TRIM28 knockdown decreased the expression of the M1‐like‐associated marker iNOS and increased the M2‐like‐associated marker Arg1, while TRIM28 overexpression reversed these trends (Figure 1F). These data confirmed that TRIM28 facilitated a shift toward an M1‐like/pro‐inflammatory macrophage marker profile and repressed an M2‐like/anti‐inflammatory marker profile of macrophages. Because macrophage activation exists along a spectrum, these findings should be interpreted as marker‐based phenotypic shifts rather than definitive binary macrophage polarization.

### 3.2 TRIM28 Influenced NLRP3 and SGT1 Expression, NLRP3 SUMOylation, and Inflammasome‐Associated Activation

**Figure 2:**
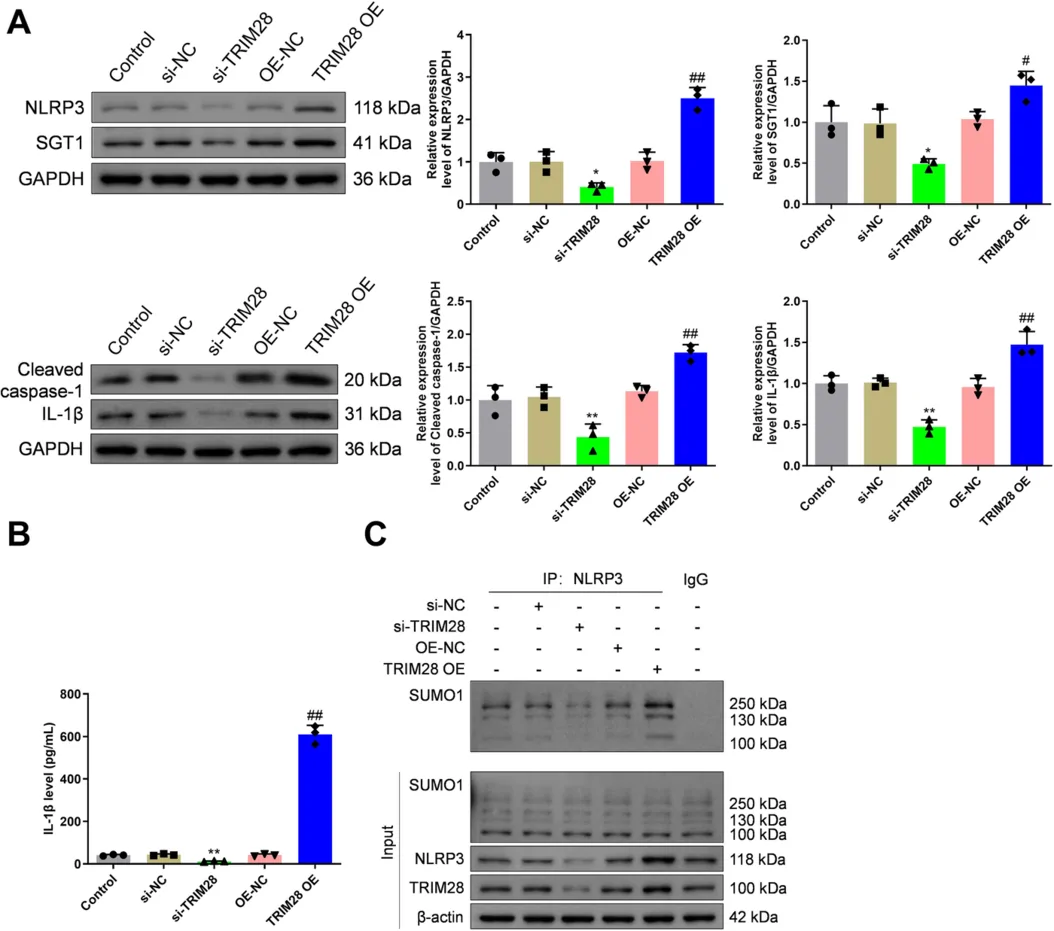
TRIM28 modulated NLRP3/SGT1 axis activation and NLRP3 SUMOylation in macrophages. (A) Western blot analysis of NLRP3, SGT1, cleaved caspase‐1, and mature IL‐1β protein expression in macrophages after TRIM28 knockdown or overexpression. GAPDH was used as the loading control. (B) ELISA detection of secreted IL‐1β levels in macrophage culture supernatants. (C) Co‐immunoprecipitation followed by Western blot analysis to detect NLRP3 SUMOylation levels. Cell lysates were immunoprecipitated with anti‐NLRP3 antibody and immunoblotted with anti‐SUMO1 antibody. Input lanes show the expression of SUMO1, NLRP3, TRIM28, and β‐actin in total cell lysates. IgG served as the negative control for IP. *p < 0.05 versus si‐NC; **p < 0.01 versus si‐NC; ^#^ *p* < 0.05; ^##^ *p* < 0.01 versus OE‐NC.

Latest research has revealed that NLRP3 exerts its activity in inducing macrophage pro‐inflammatory activation by activating its downstream molecule SGT1 [18], while TRIM28 can stabilize NLRP3 expression through SUMOylation modification [19]. We found that TRIM28 knockdown reduced the expression levels of NLRP3 and SGT1, while TRIM28 overexpression enhanced their expression. To further determine whether these molecular changes were accompanied by functional inflammasome‐associated activation, we examined cleaved caspase‐1 and mature IL‐1β expression. TRIM28 knockdown decreased cleaved caspase‐1 and mature IL‐1β levels, whereas TRIM28 overexpression increased both proteins (Figure 2A). Consistently, ELISA showed that IL‐1β secretion was reduced after TRIM28 knockdown and markedly increased after TRIM28 overexpression (Figure 2B). SUMOylation analysis demonstrated that TRIM28 overexpression significantly increased the SUMOylation level of NLRP3, while TRIM28 knockdown reduced the SUMOylation level of NLRP3 (Figure 2C). These findings indicate that TRIM28 regulates not only NLRP3 expression and SUMOylation but also downstream inflammasome‐associated readouts in macrophages.

### 3.3 TRIM28 Affected Macrophage‐Mediated HaCaT Cell Proliferation and Invasion

**Figure 3:**
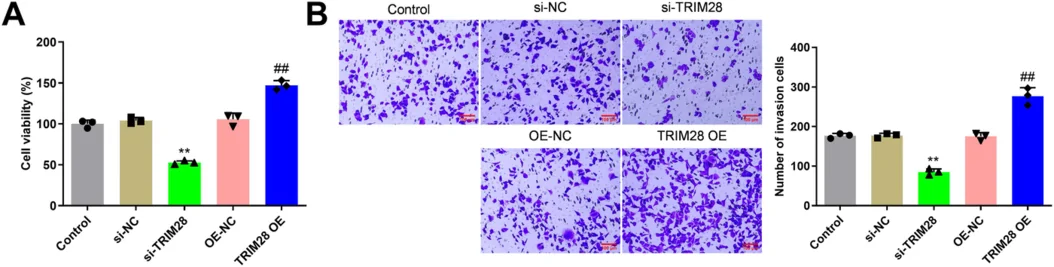
TRIM28 promoted HaCaT cell proliferation and invasion via macrophage polarization. (A) CCK‐8 assay to evaluate the viability of HaCaT cells co‐cultured with TRIM28‐modified macrophages. (B) Transwell invasion assay to quantify the number of migrated HaCaT cells after co‐culture with TRIM28‐modified macrophages. Scale bar = 100 μm. These data reflect keratinocyte responses in an in vitro co‐culture model and do not establish disease‐level relevance to psoriasis. **p < 0.01 versus si‐NC; ^##^ *p* < 0.01 versus OE‐NC.

To explore the paracrine effect of TRIM28‐modified macrophages on HaCaT cells, we established a co‐culture system using Transwell chambers. CCK‐8 assay showed that the viability of HaCaT cells co‐cultured with TRIM28 knockdown macrophages was significantly decreased to 60%, while co‐culture with TRIM28 overexpression macrophages increased cell viability to 140% (Figure 3A). Transwell invasion assay revealed a similar trend: TRIM28 knockdown in macrophages reduced the number of migrated HaCaT cells to 60%, whereas TRIM28 overexpression increased it to 180% (Figure 3B). These findings suggested that TRIM28‐induced pro‐inflammatory macrophage‐like changes promoted the proliferation and invasion of HaCaT cells, cellular behaviors that are relevant to hyperproliferative inflammatory skin responses but do not by themselves demonstrate a direct role of TRIM28 in psoriasis. Therefore, these results are presented as evidence of macrophage‐mediated regulation of keratinocyte behavior in vitro, not as evidence of psoriasis pathogenesis in vivo.

### 3.4 NLRP3/SGT1 Axis and SUMOylation Were Required for TRIM28‐Mediated Macrophage Polarization

**Figure 4:**
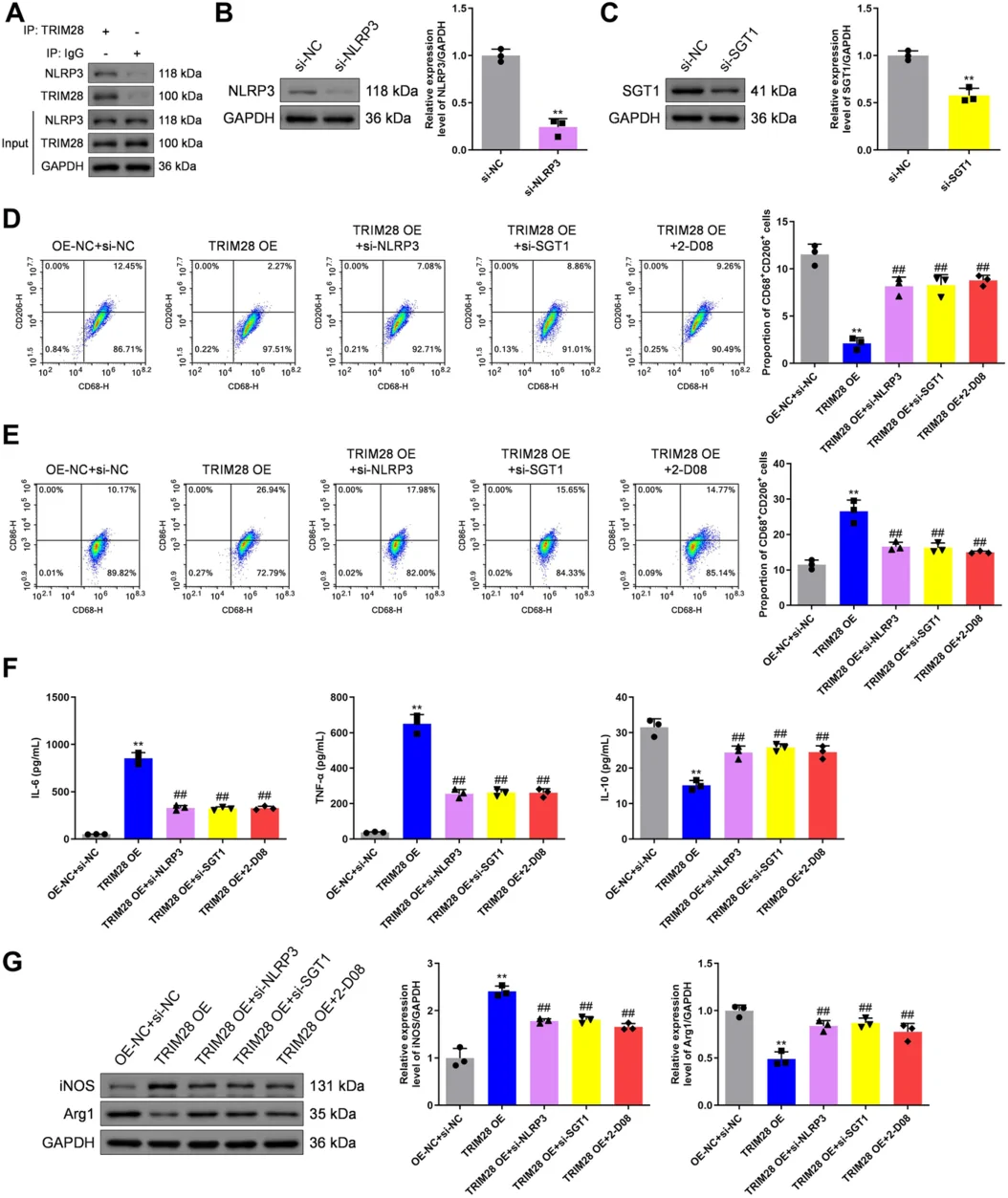
NLRP3/SGT1 axis and SUMOylation were required for TRIM28‐mediated macrophage polarization. (A) Co‐immunoprecipitation analysis of the interaction between TRIM28 and NLRP3 in macrophages. Cell lysates were immunoprecipitated with anti‐TRIM28 antibody and immunoblotted with anti‐NLRP3 antibody. IgG served as the negative control. (B) Western blot analysis of NLRP3 protein expression in macrophages transfected with si‐NLRP3 or si‐NC. (C) Western blot analysis of SGT1 protein expression in macrophages transfected with si‐SGT1 or si‐NC. GAPDH served as the loading control. **p < 0.01 versus si‐NC. (D) Flow cytometry analysis of CD68^+^CD206^+^ M2‐like macrophage proportions. (E) Flow cytometry analysis of CD68^+^CD86^+^ M1‐like macrophage proportions. (F) ELISA measurements of IL‐6, TNFα, and IL‐10 secretion levels in macrophage culture supernatants. (G) Western blot analysis of iNOS and Arg1 protein expressions, with GAPDH as the loading control. These data reflect marker‐based macrophage phenotypic shifts and should not be interpreted as definitive binary polarization states. **p < 0.01 versus OE‐NC + si‐NC; ^##^ *p* < 0.01 versus TRIM28 OE.

Co‐IP further showed that TRIM28 interacted with NLRP3 in macrophages (Figure 4A), providing additional support for a mechanistic association between TRIM28 and NLRP3 regulation. To confirm the critical role of the NLRP3/SGT1 axis and NLRP3 SUMOylation in TRIM28‐mediated biological effects, we performed rescue experiments by knocking down NLRP3 or SGT1, or inhibiting SUMOylation with 2‐D08 in TRIM28 overexpression macrophages. First, Western blot analysis validated the successful knockdown of NLRP3 and SGT1 in macrophages transfected with si‐NLRP3 and si‐SGT1, respectively (Figure 4B,C). Flow cytometry results showed that knockdown of NLRP3 or SGT1, or treatment with 2‐D08, significantly reversed the TRIM28 overexpression‐induced decrease in CD68^+^CD206^+^ M2‐like macrophages (Figure 4D) and increase in CD68^+^CD86^+^ M1‐like macrophages (Figure 4E). ELISA and Western blot assays confirmed that these interventions also reversed the changes in inflammatory cytokine secretion (IL‐6, TNF‐α, IL‐10) and the expression of iNOS and Arg1 caused by TRIM28 overexpression (Figure 4F,G). Together, these data indicate that TRIM28‐mediated pro‐inflammatory macrophage‐like phenotypic shifts are associated with TRIM28–NLRP3 interaction, NLRP3/SGT1 signaling, NLRP3 SUMOylation, and downstream inflammatory cytokine changes.

### 3.5 NLRP3/SGT1 Axis and SUMOylation Were Required for TRIM28‐Mediated Inflammasome‐Associated Activation

**Figure 5:**
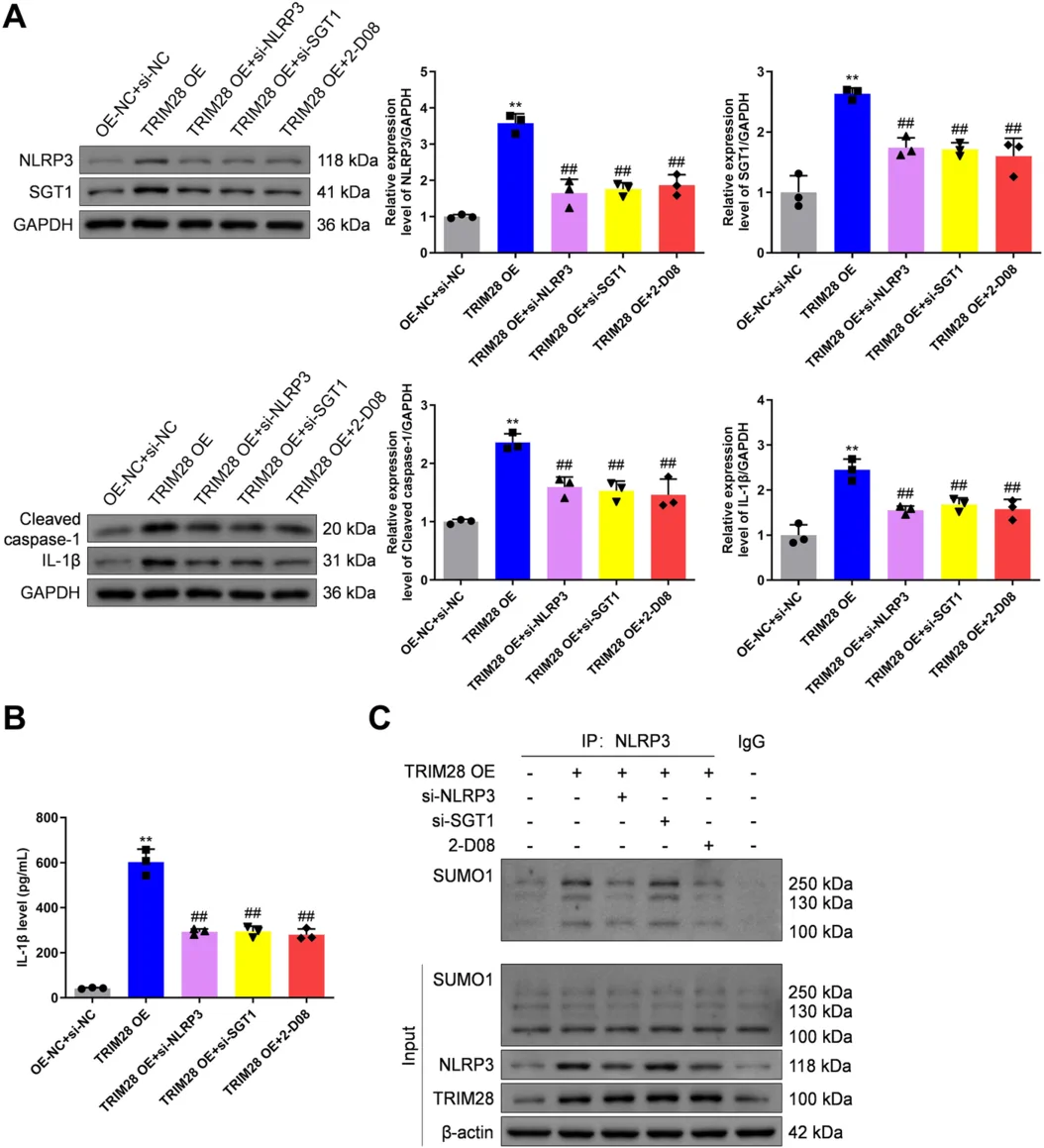
NLRP3/SGT1 axis and SUMOylation were required for TRIM28‐mediated inflammasome‐associated activation. (A) Western blot analysis of NLRP3, SGT1, cleaved caspase‐1, and mature IL‐1β protein expression in TRIM28‐overexpressing macrophages after NLRP3 knockdown, SGT1 knockdown, or 2‐D08 treatment. GAPDH was used as the loading control. (B) ELISA detection of secreted IL‐1β levels in macrophage culture supernatants. (C) Co‐immunoprecipitation followed by Western blot analysis to detect NLRP3 SUMOylation levels. Input lanes show the expression of SUMO1, NLRP3, TRIM28, and β‐actin in total cell lysates. IgG served as the negative control for IP. **p < 0.01 versus OE‐NC + si‐NC; ^##^ *p* < 0.01 versus TRIM28 OE.

To further evaluate whether the NLRP3/SGT1 axis and SUMOylation contributed to TRIM28‐induced inflammasome‐associated activation, we performed rescue experiments using NLRP3 knockdown, SGT1 knockdown, or the SUMOylation inhibitor 2‐D08 in TRIM28‐overexpressing macrophages. Western blot analysis showed that TRIM28 overexpression increased NLRP3, SGT1, cleaved caspase‐1, and mature IL‐1β levels, whereas NLRP3 silencing, SGT1 silencing, or 2‐D08 treatment partially reversed these increases (Figure 5A). ELISA further confirmed that TRIM28‐induced IL‐1β secretion was significantly reduced by NLRP3 knockdown, SGT1 knockdown, or 2‐D08 treatment (Figure 5B). In addition, Co‐IP analysis showed that the elevated NLRP3 SUMOylation induced by TRIM28 overexpression was attenuated by NLRP3 knockdown, SGT1 knockdown, or 2‐D08 treatment (Figure 5C). These results support the involvement of the NLRP3/SGT1 axis and SUMOylation in TRIM28‐mediated inflammasome‐associated signaling.

### 3.6 NLRP3/SGT1 Axis and SUMOylation Were Required for TRIM28‐Overexpressed Macrophage‐Mediated HaCaT Cell Dysfunction

**Figure 6:**
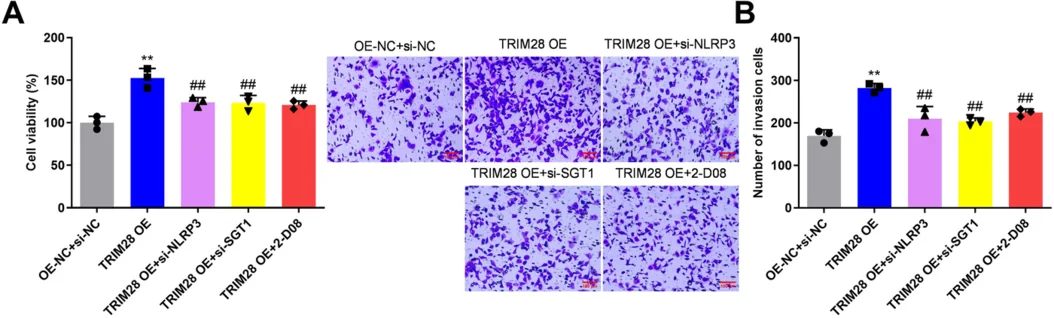
Rescue of TRIM28‐mediated HaCaT cell proliferation and invasion. (A) CCK‐8 assay to evaluate the viability of HaCaT cells co‐cultured with macrophages. (B) Transwell invasion assay to quantify the number of migrated HaCaT cells after co‐culture with macrophages. Scale bar = 100 μm. These findings support an in vitro macrophage–keratinocyte mechanism mediated by the NLRP3/SGT1 axis, but require further validation before disease‐specific conclusions can be drawn. **p < 0.01 versus OE‐NC + si‐NC; ^##^ *p* < 0.01 versus TRIM28 OE.

CCK‐8 assays indicated that the increase in cell viability induced by TRIM28 OE was partially reversed by silencing NLRP3 or SGT1, or treatment with 2‐D08 (Figure 6A). A visual representation of HaCaT cell invasion ability through staining and quantification showed that the increase in the number of invading cells induced by TRIM28 OE was partially reversed by silencing NLRP3 or SGT1, or treatment with 2‐D08 (Figure 6B).

## 4 Discussion

The present study is an in vitro mechanistic investigation showing that TRIM28 regulates macrophage polarization and macrophage‐mediated keratinocyte proliferation and invasion through the NLRP3/SGT1 axis. Macrophage activation is highly plastic and context‐dependent, and the M1/M2 framework represents only a simplified operational model rather than definitive lineage classification. Unlike T‐cell subsets, macrophage polarization lacks universally accepted lineage‐defining transcription factors; therefore, classification based on a limited marker set is inherently reductionist. We acknowledge that this work does not provide direct in vivo or clinical evidence linking TRIM28 to psoriasis. Specifically, TRIM28 expression was not examined in human psoriatic tissue, patient‐derived cells, animal models, or transcriptomic datasets, and no correlation with psoriasis severity or disease activity was assessed. Therefore, the findings should be interpreted as evidence of a TRIM28‐dependent inflammatory signaling mechanism in a cell‐line‐based macrophage–keratinocyte model, rather than as proof that TRIM28 is dysregulated or pathogenic in psoriasis.

Our findings demonstrated that TRIM28 overexpression enhanced NLRP3 inflammasome activation and SUMOylation, driving pro‐inflammatory cytokine secretion and an M1‐like/pro‐inflammatory marker profile, which subsequently exacerbated keratinocyte dysfunction. Cleaved caspase‐1, mature IL‐1β, and secreted IL‐1β were evaluated as functional readouts of inflammasome‐associated activation. TRIM28 overexpression increased cleaved caspase‐1 and mature IL‐1β protein levels as well as IL‐1β secretion, whereas TRIM28 knockdown reduced these readouts. These results support the role of TRIM28 in promoting NLRP3 inflammasome‐related signaling in macrophages. This aligns with prior evidence that TRIM family proteins, such as TRIM14 and TRIM27, are upregulated in psoriasis and contribute to disease progression by modulating inflammatory pathways like NF‐κB and IL‐6/STAT3 [20, 21]. Specifically, TRIM14‐mediated degradation of TRAF3 activates NF‐κB, while TRIM27 stabilizes IGF2BP2 to amplify IL‐6/STAT3 signaling, both exacerbating psoriatic inflammation. However, unlike these reported psoriasis‐associated TRIM proteins, the present study did not examine TRIM28 expression or activity in psoriatic tissue. Therefore, our data should not be interpreted as demonstrating that TRIM28 participates in psoriasis in vivo. Similarly, our data suggested TRIM28 acted as a critical regulator of NLRP3 inflammasome assembly, mirroring the role of HSP90β‐SGT1 in autoinflammasome activation in CAPS. The HSP90β‐SGT1 complex stabilizes NLRP3 to facilitate inflammasome formation, and its inhibition suppresses IL‐1β secretion [22]. Co‐IP showed an interaction between TRIM28 and NLRP3, suggesting that TRIM28 may regulate NLRP3 through a protein‐associated mechanism. However, whether TRIM28 directly modifies NLRP3 or recruits additional SUMOylation‐related proteins remains to be clarified. Here, TRIM28 likely potentiates this pathway by enhancing NLRP3‐SGT1 interaction and SUMOylation, though the precise structural basis requires further investigation.

Notably, our rescue experiments confirmed the indispensability of the NLRP3/SGT1 axis in TRIM28‐mediated effects. SGT1, a co‐chaperone of HSP90, is known to stabilize client proteins, and its interaction with HSP90α/β is critical for inflammasome activation [23]. Pharmacological inhibition of HSP90 disrupts SGT1 binding, attenuating NLRP3 inflammasome activity [24]. In our study, SUMOylation inhibition (via 2‐D08) or NLRP3/SGT1 knockdown reversed TRIM28‐driven M1‐like/pro‐inflammatory macrophage marker changes and keratinocyte hyperproliferation, suggesting TRIM28 may scaffold NLRP3‐SGT1‐HSP90 complexes to sustain inflammasome activity. These interventions also reduced TRIM28‐induced cleaved caspase‐1, mature IL‐1β, IL‐1β secretion, and NLRP3 SUMOylation, supporting a functional connection between TRIM28, the NLRP3/SGT1 axis, SUMOylation, and inflammasome‐associated signaling. This parallels findings in NAFLD, where HSP90 inhibition suppresses NLRP3 activation [25], and in CAPS, where HSP90β blockade ameliorates IL‐1β secretion [22]. However, our work links TRIM28 to this regulatory node in a macrophage–keratinocyte co‐culture setting, expanding its role beyond transcriptional control to include post‐translational inflammasome modulation.

The crosstalk between TRIM28 and macrophage polarization underscores its multifunctionality in immune regulation. While TRIM28 is best characterized as a transcriptional co‐repressor in cancer [26], our data reveal its pro‐inflammatory role in macrophages, akin to TRIM27's involvement in IL‐6/STAT3 activation [21]. TRIM28's ability to switch from a repressor to an activator, as seen in HIV transcription [27], may explain its context‐dependent effects. In this in vitro system, TRIM28 likely promotes NLRP3 SUMOylation to stabilize the inflammasome, analogous to how SUMOylation of TRIM28 itself modulates its interaction with KRAB‐ZNF proteins [28]. The SUMOylation of NLRP3, however, remains underexplored. Prior studies show that TRIM28 SUMOylation is critical for its repressive function [29], but our results suggested it may also license NLRP3 activation. This dichotomy warrants further study, particularly whether TRIM28 directly SUMOylates NLRP3 or recruits SUMO ligases. Additionally, the interplay between TRIM28 and other TRIM proteins (e.g., TRIM32, which enhances EMT in fibroblasts [30]) could synergize to amplify inflammatory skin‐associated cellular responses.

## 5 Study Limitations

Despite these insights, our study has limitations. Most importantly, the present work was performed entirely in simplified in vitro systems using PMA‐differentiated THP‐1 cells and immortalized HaCaT keratinocytes. These models cannot fully recapitulate the cellular diversity, tissue architecture, cytokine networks, and immune microenvironment of psoriatic skin. We did not examine TRIM28 expression in human psoriatic lesions or non‐lesional skin, did not perform in vivo validation in psoriasis‐like animal models, did not analyze patient‐derived samples, and did not assess correlations between TRIM28 and disease severity or established psoriasis transcriptomic signatures. Therefore, our data cannot demonstrate that TRIM28 is dysregulated in psoriasis or that it functions as a driver of psoriasis pathogenesis in vivo. Therefore, TRIM28 should not be considered a psoriasis‐specific therapeutic target or a validated psoriasis‐pathogenesis molecule based on the present data. The translational relevance of this mechanism remains speculative until supported by human tissue validation, patient‐derived cellular models, disease transcriptomic analyses, and appropriate in vivo studies. Future work should validate these findings in animal models, such as imiquimod‐induced psoriasis, to assess TRIM28's role in vivo. Second, while we identified NLRP3 SUMOylation as a key mechanism, the specific E3 SUMO ligase involved remains unknown. TRIM28 itself lacks SUMO ligase activity, suggesting intermediary proteins like PIAS1 may bridge this interaction. Although Co‐IP supports an interaction between TRIM28 and NLRP3, the structural domains or intermediary proteins responsible for this interaction remain undefined. Third, the clinical relevance of TRIM28 in psoriasis patients needs confirmation. Although TRIM27 and METTL14 are elevated in psoriatic skin, TRIM28 expression in patient samples was not examined. Finally, the therapeutic potential of targeting TRIM28 or its downstream effectors (e.g., HSP90 inhibitors) requires evaluation, considering their broad roles in other diseases. In addition, macrophage polarization was assessed using a limited marker panel, including CD86/CD206, iNOS/Arg1, and IL‐6/TNF‐α/IL‐10. These markers can suggest relative M1‐like or M2‐like phenotypic shifts, but they cannot fully define macrophage states. A more comprehensive characterization using broader surface and intracellular marker panels, transcriptomic profiling, single‐cell RNA sequencing, multiplex flow cytometry, and dimensionality‐reduction approaches such as t‐SNE or UMAP would provide a more realistic assessment of macrophage heterogeneity and activation states. Therefore, our conclusions regarding macrophage polarization should be interpreted cautiously as marker‐based inflammatory phenotypic changes rather than definitive macrophage subtype classification. In addition, although inflammasome‐associated activation was evaluated by cleaved caspase‐1, mature IL‐1β, and secreted IL‐1β, the study did not fully define all molecular steps of inflammasome assembly, such as ASC speck formation or direct caspase‐1 enzymatic activity. Thus, the inflammasome‐related conclusions should be interpreted as evidence of inflammasome‐associated signaling rather than complete characterization of inflammasome activation. Moreover, the functional assays were based on HaCaT proliferation and invasion in a Transwell co‐culture system, which may not fully represent primary keratinocyte behavior in a native skin microenvironment.

## 6 Future Research Directions

Future studies should extend the present findings in several directions. First, TRIM28 expression and localization should be examined in human psoriatic lesional and non‐lesional skin, ideally in parallel with clinical disease severity and established inflammatory markers, to determine whether TRIM28 is associated with psoriasis at the tissue level. Second, patient‐derived macrophages, primary keratinocytes, and more physiologically relevant skin models, such as three‐dimensional organotypic cultures, should be used to validate the TRIM28–NLRP3/SGT1 axis in cellular systems that better reflect the human skin microenvironment. Third, in vivo studies using psoriasis‐like animal models, such as imiquimod‐induced psoriasiform dermatitis, are needed to evaluate whether modulation of TRIM28 alters cutaneous inflammation, keratinocyte hyperproliferation, and macrophage activation in a tissue context. Fourth, broader macrophage phenotyping using multiplex flow cytometry, transcriptomic profiling, single‐cell RNA sequencing, and dimensionality‐reduction analyses may help define the macrophage states regulated by TRIM28 more accurately. Fifth, the molecular mechanism by which TRIM28 regulates NLRP3 SUMOylation requires further investigation, including identification of the relevant SUMO E3 ligase, mapping of the interaction domains between TRIM28 and NLRP3, and assessment of ASC speck formation and caspase‐1 enzymatic activity. Finally, the potential therapeutic value and safety of targeting TRIM28 or downstream NLRP3/SGT1‐related signaling should be evaluated in disease‐relevant experimental models before any translational application is considered.

In conclusion, our study identifies TRIM28 as a regulator of macrophage M1‐like/pro‐inflammatory phenotypic shifts and macrophage‐mediated keratinocyte proliferation and invasion through activation of the NLRP3/SGT1 axis in vitro. These findings provide a mechanistic basis for future investigation of TRIM28 in inflammatory macrophage–keratinocyte interactions. However, additional validation in human psoriatic tissues, patient‐derived samples, transcriptomic datasets, and in vivo models is essential before TRIM28 can be considered directly involved in psoriasis pathogenesis or proposed as a psoriasis‐specific therapeutic target. Thus, the main contribution of this study is mechanistic rather than translational: it defines a TRIM28–NLRP3/SGT1‐related pathway in a simplified macrophage–keratinocyte co‐culture system, while its relevance to psoriasis remains to be established.

## Author Contributions

Conception and design of the research: Zhe Gao and Xin Zhang. Acquisition of data: Zhe Gao and Xin Zhang. Analysis and interpretation of data: Juan Wang. Statistical analysis: Ni Yang. Obtaining funding: Zhe Gao. Drafting the manuscript: Zhe Gao. Revision of manuscript for important intellectual content: Xin Zhang.

## Use of AI‐Assisted Software

The authors acknowledge that AI‐assisted software was used only for language polishing and grammar improvement during the preparation of this manuscript. AI was not used for study design, data collection, data analysis, data interpretation, figure preparation, or generation of scientific content. The authors take full responsibility for the content of the manuscript.

## Ethics Statement

The authors have nothing to report.

## Conflicts of Interest

The authors declare no conflicts of interest.

## Data Availability

Data will be made available on request.
